# When *little brain* goes to school: Impact of pedagogy on cerebellar peduncles’ development

**DOI:** 10.1016/j.dcn.2026.101748

**Published:** 2026-05-25

**Authors:** Gabriel Girard, Mathilde Gaujard, Camille Grosjean, Elda Fischi-Gomez, Solange Denervaud

**Affiliations:** a Signal Processing laboratory 5, Swiss Federal Institute of technology (EPFL), Lausanne 1015, Switzerland; b Department of Radiology, Lausanne University Hospital and University of Lausanne (CHUV-UNIL), Lausanne, Switzerland; c CIBM Center for Biomedical Imaging, Switzerland; d MRI imaging and technology, Polytechnical School of Lausanne, Swiss Federal Institute of Technology Lausanne (EPFL), Lausanne 1015, Switzerland

**Keywords:** Cerebellum, Neurodevelopment, Diffusion MRI, Learning environment, White matter plasticity

## Abstract

The cerebellum plays a central role in motor coordination, cognition, and learning, yet its developmental sensitivity to students’ everyday learning environments remains poorly understood. Here, we examined whether schooling context is associated with differences in the developmental trajectories of cerebellar white matter pathways. Using multi-shell diffusion-weighted Magnetic resonance imaging (MRI), we quantified microstructural properties of the inferior (ICP), middle (MCP), and superior cerebellar peduncles (SCP) in 88 typically developing students aged 4–16 years, including a longitudinal subsample of 34 participants reassessed after approximately three years. Across tracts, higher intra-cellular volume fraction (ICVF) and lower mean diffusivity (MD) were associated with better fluid intelligence, selective attention, and working memory, indicating functional relevance of cerebellar microstructure for cognitive development. Age was a strong predictor of MD and ICVF across all cerebellar peduncles, consistent with ongoing maturation throughout childhood and adolescence. Crucially, significant ageXschooling interactions emerged for fractional anisotropy (FA) within the ICP and SCP, suggesting that the pace of cerebellar white matter maturation differs as a function of school experience. Longitudinal analyses provided convergent, trend-level support for these patterns, with traditionally schooled students showing numerically larger microstructural change in the right ICP (MD) and left ICP and SCP (FA). Classification analyses yielded modest discrimination between schooling contexts, indicating that cerebellar microstructure alone does not robustly encode educational background. Together, these findings provide evidence that cerebellar white matter development follows experience-sensitive trajectories, highlighting schooling as a meaningful environmental factor contributing to individual variability in neurodevelopment.

## 1 Introduction

The cerebellum plays a central role in integrating motor commands with sensory feedback, supporting adaptive learning, coordination, and prediction (Firouzi et al., 2024, Manto et al., 2012, Popa et al., 2016). Through its extensive connectivity with the cerebral cortex, brainstem, spinal cord, and peripheral sensory systems, the cerebellum functions as a key hub for detecting discrepancies between intended and actual outcomes. This error-based learning mechanism is fundamental not only for motor coordination but also for implicit cognitive processes and perceptual learning (Baumann et al., 2015, Ma et al., 2021, Seidler et al., 2013, Van Overwalle et al., 2020). Most of these functions are intensively recruited and refined during the school years (Mastrangelo et al., 2024). However, despite the cerebellum’s prolonged developmental trajectory, its sensitivity to different schooling experiences has not yet been systematically investigated.

Cerebellar development follows a highly orchestrated and extended sequence of maturational processes. Between 20 and 40 weeks of gestation, the cerebellum undergoes a period of rapid growth that exceeds that of any other cerebral structure (Stoodley and Limperopoulos, 2016). Postnatally, cerebellar maturation continues throughout childhood and adolescence, likely reflecting ongoing cellular differentiation, axonal growth, and white matter proliferation. Total cerebellar volume follows an inverted U-shaped developmental trajectory, peaking at age 11.8 years in females and 15.6 years in males (Tiemeier et al., 2010). Similarly, the volume and microstructure of the cerebellar peduncles continue to increase from birth to age 30 (Olson et al., 2023). Once fully developed, the cerebellum contains approximately five times more neurons than the cerebrum, underscoring its computational capacity (Herculano-Houzel, 2009).

Beyond its well-established role in motor control (Firouzi et al., 2024, Manto et al., 2012, Matsumura et al., 2004), the cerebellum is now increasingly recognized as a critical substrate for higher-order cognitive functions, including visuo-spatial processing, attentional control, working memory, and language (Baumann et al., 2015, Sokolov, 2018, Zhang et al., 2023). Notably, the role of the cerebellum is especially pronounced during development and skill acquisition (Ziegler and Ackermann, 2017). Large cerebellar lesions sustained during childhood can lead to profound impairments in executive function, language, spatial cognition, and affective regulation, with more severe and long-lasting deficits than those observed following lesions in adulthood (Beuriat et al., 2020). These impairments are collectively described in cerebellar cognitive affective (Schmahmann) syndrome (Hoche et al., 2018). Importantly, cerebellar dysfunction is not confined to overt lesions. Subtle alterations in cerebellar processing have been implicated in neurodevelopmental disorders that affect learning and cognition. For example, dyslexia, where cerebellar deficits may disrupt the automation of reading skills and phonological processing (Stoodley and Stein, 2013). Similarly, attention-deficit/hyperactivity disorder has been associated with impairments in cerebellar-related attentional regulation and motor control (Bledsoe et al., 2011, Cundari et al., 2023). Together, these findings underscore the cerebellum as a pivotal structure supporting cognitive and learning processes across development (Mastrangelo et al., 2024).

Consistent with its role in adaptive learning, the cerebellum exhibits a high degree of experience-dependent plasticity. Neuroimaging studies indicates that cognitive demands and training modulate cerebellar structure and function. For example, Bonnet et al. (2009) demonstrated that in healthy young adults, higher levels of education were associated with greater cerebellar activation and reduced engagement of cortical regions, such as the medial prefrontal and inferior parietal cortex, during an fMRI Go/No-go task. In highly educated participants, the cerebellum assumes the more automated parts of the attentional demands required to perform this cognitive task to spare cortical regions (Bonnet et al., 2009). This sensitivity to experience is also found following sensorimotor training. After eight weeks of drumming sessions, the cerebellum changed significantly in the inferior cerebellar peduncle’s gray and white matter microstructure, compared with an age-matched control group of non-musicians (Bruchhage et al., 2020). These cerebellar plastic changes were related to changes in cortical thickness, suggesting an interaction between cerebellar learning and cortical structures activated by cerebellar pathways (Bruchhage et al., 2020). As part of the cognitive skills its supplies, the posterior cerebellum is involved in social cognition, and more specifically in the interpretation of goal-directed actions through the movements of other people (“social mirroring”), as well as in the social understanding of the mental states of different individuals (“social mentalizing”). The cerebellum contributes to learning and understanding sequences of social actions and thus facilitates social cognition by supporting optimal predictions about imminent or future social interaction and cooperation (Van Overwalle et al., 2020). Across childhood, such motor, cognitive, and social experiences are densely embedded within schooling environments. Yet, to date, no study has examined whether different educational contexts are associated with differential cerebellar development

In Switzerland, traditional schooling typically involves teacher-led instruction delivered to same-age classrooms, structured schedules with successive lessons, and limited opportunities for movement outside designated periods such as recess and sports classes (www.plandetudes.ch). Feedback is primarily provided through grades and formal assessments. In contrast, alternative pedagogical approaches such as Montessori education differ markedly in these dimensions. Montessori classrooms are characterized by mixed-age groups, peer-to-peer learning, self-corrective materials, free movement during learning periods, and extended uninterrupted work cycles that promote autonomy and sustained engagement (Marshall, 2017). Recent meta-analyses and empirical studies indicate that these distinct pedagogical frameworks are associated with differences in children’s behavior and cognitive development (Courtier et al., 2021, Demangeon et al., 2023, Randolph et al., 2023).

Specifically, Montessori education has been linked to enhanced language abilities (i.e. letter-word identification, phonological decoding ability) in early childhood (Lillard and Else-Quest, 2006), more advanced emotion regulation and emotion recognition skills (Denervaud et al., 2020d, Dereli İman et al., 2019). Also, children enrolled in Montessori classes had a more flexible organization of semantic memory with concepts more interconnected and enriched, than in children enrolled in traditional classes (Denervaud et al., 2021), and underlying brain structures have also been observed (Schetter et al., 2023). For attentional skills too, previous studies have highlighted a positive impact of Montessori education (Aicoboaie et al., 2025, S, 2015). Montessori-schooled children seem to experience learning environments that place greater demands on adaptive capacities (Denervaud et al., 2024, Denervaud et al., 2020a, Denervaud et al., 2020c, Denervaud et al., 2019). A critical and underexplored question concerns the mechanisms through which pedagogy might shape cerebellar development. These schooling environments may differ markedly in the extent to which they engage cerebellar-relevant processes. Specifically, the cerebellum plays a central role in sensorimotor integration, coordinating movement with sensory feedback and adapting behavior based on prediction errors (Popa et al., 2016). Environments that involve frequent physical manipulation of materials, embodied learning, and self-directed movement, as in Montessori classrooms, are hypothesized to recruit these sensorimotor circuits more consistently than teacher-led, sedentary instruction. In addition, the cerebellum is sensitive to timing and sequencing demands, which differ substantially between structured versus flexible learning contexts (Ivry and Schlerf, 2008). Finally, social coordination and peer-to-peer learning, which are central to mixed-age Montessori classrooms, engage cerebellar circuits implicated in social prediction and mentalizing (Van Overwalle et al., 2020). These mechanistic considerations motivate a direct comparison of cerebellar development across contrasting schooling contexts. Together with preliminary findings in cerebellar differences (Zanchi et al., 2024), and in line with the differences observed in competences that rely on the cerebellum (e.g., motor skills, memory, attention, social cognition), we hypothesize that developmental trajectories of cerebellar peduncles are sensitive to schooling experience.

Structurally, the cerebellum is integrated into large-scale neural networks via three major white matter tracts, known as the cerebellar peduncles, which serve as critical pathways linking the cerebellum to the brainstem and other cortical and subcortical structures. Among them, the superior cerebellar peduncle (SCP) constitutes the principal efferent output of the cerebellum, transmitting signals to cortical regions involved in cognitive control (Okugawa et al., 2006) and motor planning (Israely et al., 2025). The middle cerebellar peduncle (MCP) conveys pontine input through distinct sub pathways, primarily associated with both higher cognitive and sensorimotor functions (Re et al., 2017, Travis et al., 2015). This is reflected in children with ADHD who have impaired anatomical connectivity of the middle cerebellar peduncle (Parkkinen et al., 2024). The inferior cerebellar peduncle (ICP) primarily carries afferent sensory information from the spinal cord and brainstem and supports the integration and storage of motor and posture information (Bruchhage et al., 2020), including maintaining balance and posture (Re et al., 2017).

These pathways can be interrogated using Diffusion-Weighted Magnetic Resonance Imaging (DW-MRI), a non-invasive technique that characterizes white matter microstructure by modeling water diffusion within tissue (Basser and Pierpaoli, 1996). Diffusion-derived metrics such as fractional anisotropy (FA), mean diffusivity (MD), and intracellular volume fraction (ICVF) provide complementary information about fiber organization, tissue density, and cellular composition. FA is a scalar value that indexes the degree of directional preference in the water diffusion model, informing about the microstructural integrity and organization of white matter (Beaulieu, 2002). MD evaluates the overall rate of water diffusion, informing about the overall tissue density and neurodevelopmental change (Lebel et al., 2012). Finally, the ICVF is a measure of cellular density and informs about the proportion of intracellular space within the tissue (Debiasi et al., 2025). From a developmental point of view, these metrics have been well characterized for the cortex. Developmental studies have shown that FA and MD follow prolonged, non-linear trajectories across childhood and adulthood, while tract volume increases with age but does not strongly account for these diffusion changes (Lebel et al., 2012).

In the present study, we examined whether the developmental trajectories of the three main cerebellar peduncles differ as a function of schooling experience. Using cross-sectional and longitudinal diffusion MRI data, we compared students attending traditional or Montessori schools to investigate potential experience-dependent modulation of cerebellar white matter development. We hypothesized that cerebellar peduncle microstructure would exhibit age-related maturation and that the pace of this maturation would differ across educational contexts, reflecting the cerebellum’s sensitivity to experiential factors embedded in schooling environments.

## 2 Method

### 2.1 Participants

To study neurodevelopment in relation to schooling experience, students attending different pedagogical systems were recruited as part of a large-scale research project. Inclusion criteria were: (i) enrollment in either a traditional or Montessori school, (ii) age between 4 and16 years, and (iii) absence of parent-reported neurodevelopmental or learning disorders.

Eighty-eight students participated in the study (mean age = 9.14, SD = 2.20, 43 girls; Table 1). Of these, 42 participants attended Montessori schools (mean age = 9.08, SD = 2.13, 18 girls) and 46 participants attended traditional schools (mean age = 9.19, SD = 2.29, 25 girls). A subset of 34 participants returned for a follow-up assessment approximately three years later (mean interval = 2.86 years, SD = 0.23): 15 were Montessori-schooled (mean age at T1 = 8.91, SD = 1.51; 6 girls) and 19 were traditionally schooled (mean age at T1 = 9.48, SD = 2.02; 9 girls). At T1, these participants ranged from 5.9 to 12.8 years (mean age = 9.23, SD = 1.81). At T2, participants ranged from 8.4 to 15.7 years (mean age = 12.09, SD = 1.88). The two groups did not differ significantly in age (t = 0.94, p = .355), sex (χ² = 0.18, p = .667), or SES (t = -0.83, p = .413) at T1. Full descriptive statistics and between-group comparisons for the longitudinal subgroup are provided in Supplementary Table S1. To assess representativeness, longitudinal participants were compared to non-returners on key baseline variables (age, SES, fluid intelligence, selective attention, working memory, and diffusion metrics). No significant differences were found between the two groups on any variable (all p > .05), indicating that the longitudinal subsample was broadly representative of the full baseline sample.

**Table 1:** **T-test statistics for demographic and cognitive variables** between the Montessori (M) and traditionally-schooled (T) participants.

**Variable**	**Mean T**	**Mean M**	**Standard-Error T**	**Standard-Error M**	**T-test related p-value**
**Socioeconomic Status**	3.03	3.03	0.0809	0.0726	0.992
**Pedagogy Interest**	1.89	2.10	0.115	0.098	0.201
**Activities in Family**	8.66	9.02	0.312	0.248	0.366
**Parental Worry About Child’s Performance**	**9.07**	**5.12**	**0.276**	**0.478**	**< 0.001**
**Anxiety**	33.3	29.1	2.24	2.43	0.211
**Suppression Emotion**	17.4	17.1	0.788	0.868	0.805
**Cognitive Reappraisal**	7.59	7.5	0.58	0.535	0.909
**Fluid Intelligence**	31.9	32.8	0.615	0.554	0.295
**Selective Attention**	0.912	0.894	0.0178	0.0253	0.575
**Working Memory**	0.861	0.82	0.0211	0.0391	0.36
**Cognitive Flexibility**	0.741	0.723	0.0239	0.0315	0.642
**Divergent Thinking**	**6.16**	**8.95**	**0.582**	**0.699**	**0.008**
**Convergent Thinking**	**3.52**	**4.86**	**0.206**	**0.25**	**< 0.001**

Participants gave oral consent, and their parents provided written consent. Participants received a gift voucher or a book on neuroscience. The present study was conducted per the Declaration of Helsinki, and the Commission d′Ethique Romande approved the study protocol (CER-Vaud).

### 2.2 Experimental design

Participants were invited to the CHUV (Centre Hospitalier Universitaire Vaudois) radiology laboratory (Lausanne, Switzerland). The experimental session consisted of two parts, the order of which was randomized between participants: a behavioral session outside the scanner to collect neuropsychological and demographic data, and an MRI session to collect neuroimaging data.

### 2.3 Demographic and cognitive variables

To counteract a potential selection bias due to the different school systems of the participants, demographic variables were collected, as well as cognitive ability measures where the participant was further asked to perform several tasks of fill in questionnaires. First, parents were asked about their:

– *Socioeconomic status (SES)* is a measure of the participants' family’s wealth level and social status. This status was assessed using a questionnaire (Genoud, 2011) concerning the educational (from 0 to 4) and occupational (from 0 to 4) levels of both parents or one (in case of single-parent family situation). The average of the responses was calculated to form the final SES score. The highest score (4/4) indicates a high family socio-economic level (Table 1).
– *Pedagogy interest* in pedagogy is a measure of the importance participants' families attach to their child's educational development. This score was assessed using a questionnaire (3 questions) on parental interest in education and pedagogy (e.g., number of books on education they have read). Answers were summed. The highest score (3/3) indicates greater parental interest in child development and greater knowledge about pedagogy (Table 1).
– *Worry about performance* is a measure of the importance parents attach to their child’s academic performance. This score was evaluated on a 5-point scale (from never to always). The highest score (5/5) reflects parents who are highly concerned about their child’s performance at school.
– *Family activities* is a measure that represents the moments of sharing that take place in the family. This score was measured using a questionnaire (from 0 to 11) with suggested activities. Parents were asked to tick the activities they did as a family (e.g., drawing, singing or cooking together). Answers were summed. The highest score (11/11) indicates many shared family activities.
– Second, each participant was asked to perform tasks and fill in questionnaire about:
– *Fluid intelligence (FI)* is a measure of the participant’s ability to analyze, think logically and solve problems in novel circumstances. This measure was assessed by the black and white paper version of the Raven’s Progressive Matrices Test (PM-47) (Raven, 2016). The 15-minute test consisted of 3 sets of 12 matrices, each of which was missing one part, and the participant had to complete each matrix with one of the 6 or 8 choices offered. Although often used as an index of fluid intelligence, this test more specifically measures abstract and inductive reasoning abilities. Each correct item granted 1 point. The sum of all correct answers gave the fluid intelligence score, with the maximum score being 36. Raw scores were used in the analyses, as age was included as a continuous covariate in the statistical models, and standardized norms for the PM-47 are not consistently available across the full age range studied.
– *State anxiety (Anxiety)* is a measure of the anxiety felt by the participant at the time of answering the questionnaire. It is measured using a self-report questionnaire with six items (from strongly disagree to strongly agree) (Marteau and Bekker, 1992). Responses were summed and transformed into percentages. The highest score (100) indicates that the participant feels very anxious.
– *Creativity* measures participants’ ability to deviate from instructions to come up with innovative ideas. Creativity can be measured through divergent and convergent thinking skills separately. Here, we assessed divergent thinking, the participant was asked to produce as many different drawings as possible from an abstract form within a predefined timeframe of 5 min. To make a score, the sum of each valid drawing was applied, i.e., a single concrete drawing in which the given initial shape was used. There was no maximum in this task. Convergent thinking was measured using the standardized nonverbal task from the Evaluation of Potential Creativity (EpoC) battery (Lubart et al., 2019). The participant was asked to create a drawing incorporating at least three of eight abstract shapes (for example, an oval, triangle or square) within 10 min. To assess convergent thinking, each drawing was rated on a scale from 1 (not very creative drawing) to 7 (very creative drawing) by three independent raters, taking into account shape integration (i.e., shapes were used appropriately in the drawing), originality (i.e., the participant drew something different from other participants) and storytelling (i.e., the participant was able to share a story through his or her drawing). Scores were averaged, and high scores indicate highly creative performance (maximum 7).
– *Emotion regulation* is a measure of how well a participant expresses or controls their feelings in different situations. This score is evaluated using the Emotion Regulation Questionnaire (ERQ) (Gross and John, 2003), a 10-item questionnaire assessing frequency of use of two common emotion regulation strategies: cognitive reappraisal and expressive suppression. Separate scale scores are derived for these two regulation strategies. All items are answered on a 10-point Likert scale, ranging from 1 (strongly disagree) to 5 (strongly agree), with higher scores indicating higher usage of that strategy.
– *Selective attention* and *Cognitive flexibility* are measures of students’ ability to adapt to non-routine situations. These metrics were assessed using the Flanker fish task (Eriksen and Eriksen, 1974), a child-friendly version of the Flanker task, where arrows are replaced by fish, was used to measure selective attention and a cognitive flexibility score. These measures are derived from the accuracy rate. In this experiment, the participant had to press on a button according to the fish orientation (replacing the arrow) across three different blocks. The rules moved from the first block (focus on the fish in the center of a line of five blue fish - 17 trials) to the second block (focus on the four fish flanking the central fish, all pink - 17 trials). The last block randomly mixed the two instructions (line of five blue fish or five pink fish for 45 trials). Response time (RT) was limited to 2000 ms for children under 6 and 1500 ms for older students. Trials with a valid RT (within 2 SDs) with correct answers were computed as follows: for selective attention, the number of correct answers across the first block were summed. For cognitive flexibility, the number of correct answers across the second block were summed. High scores indicate good attentional performance and cognitive flexibility.
– *Working memory* is a measure of participant’s ability to store and manipulate short-term information. The score was measured using ascending digit repetition tasks (up to 6 years) or digit and letter repetition tasks (over 6 years) (item from the WISC-IV). The participant listened to and memorized a series of mixed letters and digits, repeating them in ascending order (i.e., mentally reorganizing the information). The score was standardized for age (i.e., a higher score indicates greater working memory capacity).

### 2.4 Neuroimaging data

#### 2.4.1 Acquisition

MRI data was acquired using a 3.0 T Siemens Prisma scanner equipped with a 64-channel head coil at the CIBM Centre for Biomedical Imaging, Lausanne University Hospital (CIBM-CHUV). Prior to entering the scanning session, participants were trained with a mock scanner. Then, each participant underwent a scanning session that included three distinct acquisition protocols.

First, high-resolution structural imaging was performed using a 3D T1-weighted MPRAGE (Magnetization Prepared Rapid Gradient Echo) sequence, lasting five minutes. The acquisition parameters were: repetition time (TR) = 2000 ms, echo time (TE) = 2.47 ms, inversion time (TI) = 900 ms, field of view (FOV) = 208 × 256 × 256 mm³ , flip angle = 8°, and voxel size = 1 × 1 × 1 mm³ . Second, Diffusion-Weighted Magnetic Resonance Imaging (DW-MRI) was conducted over 13 min with the following settings: TR = 5100 ms, TE = 80 ms, FOV = 233.6 × 233.6 × 144 mm³ , flip angle = 90°, voxel size = 1.6 × 1.6 × 1.6 mm³ , acceleration factor PE (phase-encoding) = 3, and multiband acceleration factor = 2. Diffusion encoding was performed across four shells (b = 700, 1000, 2000, and 3000 s/mm²) with 6, 20, 45, and 66 diffusion-weighted directions, respectively, yielding 137 volumes in total, complemented by 11 non-diffusion-weighted (b = 0) volumes. Finally, a functional MRI acquisition was performed but is not included in this study.

Participants watched a movie of their choice to ensure comfort during the scans. The entire imaging session lasted approximately 60 min, completed without sedation or injections. Participants wore headphones while watching their movie to reduce noise discomfort, and foam cushions were placed around their ears to minimize head movement.

#### 2.4.2 Data processing

**Fig. 1:**
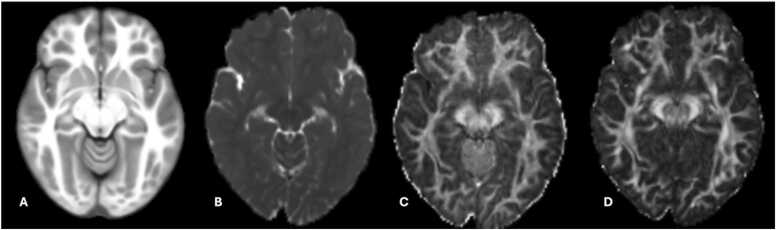
Exemplary (A) T1 weighted, (B) MD, (C) Intra, and (D) FA data.

**Fig. 2:**
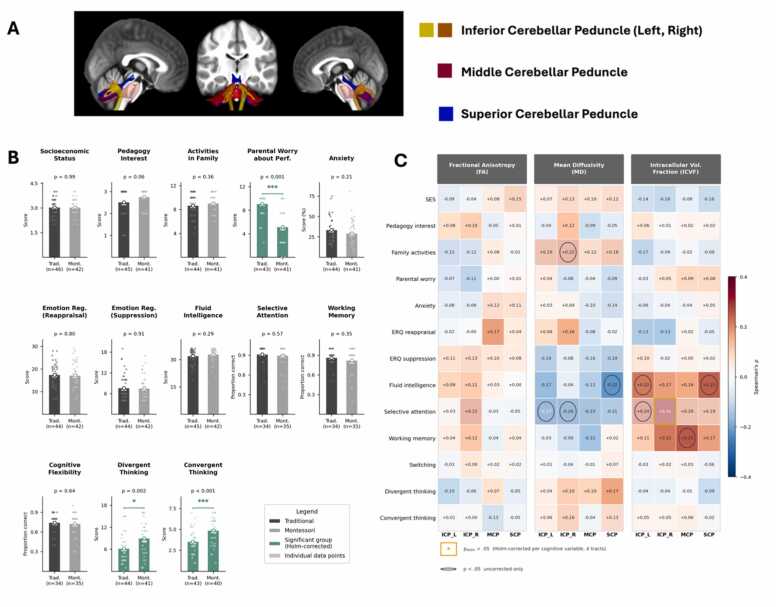
**Cerebellar peduncles, demographic and cognitive comparisons, and correlation matrices.** (A) Representative cerebellar peduncles of the left hemisphere from a participant. Tracts are overlaid on individual anatomical MPRAGE T1 images. The left and right inferior cerebellar peduncles (ICP) are shown in yellow, the middle cerebellar peduncle (MCP) in red, and the superior cerebellar peduncle (SCP) in blue. (B) Bar plots comparing demographic and cognitive variables between Montessori- and traditionally schooled participants, with significant differences highlighted in green. (C) Correlation heatmaps illustrating Spearman's ρ between demographic and cognitive variables and three diffusion metrics, fractional anisotropy (FA), intracellular volume fraction (ICVF), and mean diffusivity (MD), within the four cerebellar peduncles (ICP_L, ICP_R, MCP, SCP; n = 88). Gold-bordered cells indicate associations surviving Holm’s correction per cognitive variable (4 tracts); cells marked ○ reached uncorrected p < .05 only.

Raw diffusion-weighted images were preprocessed following recommendations of the TractoFlow pipeline (Theaud et al., 2020), which includes: Gibbs ringing correction (Tournier, 2019), susceptibility-induced distortion correction using reversed phase-encoding b= 0 images (Andersson et al., 2003), eddy current correction, and inter-volume motion correction (Andersson and Sotiropoulos, 2016). Data quality was assessed through visual inspection of raw and preprocessed images, and participants were excluded if more than 10% of volumes were affected by signal dropout or gross motion artefacts. The mean framewise displacement was inspected across age groups and pedagogy groups to verify the absence of systematic motion-related bias; no significant differences were found. No participant was excluded on quality criteria. Probabilistic tractography was performed using MRtrix3 (Tournier et al., 2019) with the iFOD2 algorithm. Fiber orientation distributions were estimated using multi-shell multi-tissue constrained spherical deconvolution (Jeurissen et al., 2014). We extracted the mean values of two diffusion tensor-derived metrics (FA and MD; Tournier et al., 2019) and the intracellular volume fraction (ICVF) from multi-compartment modelling (Kaden et al., 2016a), yielding three diffusion measures for each tract, as displayed in Figs. 1 and 2A: (i) FA that reflects the directional coherence of water diffusion along white matter tracts and is commonly used as an index of white matter integrity; (ii) MD which quantifies the average magnitude of diffusion within a voxel and thus reflects tissue density and integrity; (iii) ICVF which represents the fraction of diffusion signal arising from water confined within the intra-cellular space, providing an estimate of the density of cellular structures (Jelescu and Budde, 2017, Kaden et al., 2016b, Van Hecke et al., 2016).

T1-weighted images were registered to diffusion space using the mean b = 0 and FA images with ANTs (Avants et al., 2011) following recommendations of Theaud et al. (2020) (Theaud et al., 2020). The IIT Human Brain Atlas v.5.0 (Qi and Arfanakis, 2021) was subsequently registered to each subject’s diffusion space to extract streamline density maps of four cerebellar tracts: the left and right ICP, the MCP, and the SCP. The SCP was treated as a single bilateral tract, consistent with the atlas definition in IIT Human Brain Atlas v.5.0, where the SCP is represented as a midline structure reflecting the decussation of cerebellar efferent fibers at the level of the mesencephalon, which makes hemisphere-specific reconstruction anatomically unreliable with tractography. For each tract, diffusion measures were computed as streamline-weighted means across all corresponding voxels.

### 2.5 Statistical analysis

All analyses were done with Jamovi (version 2.4) Computer Software and R (version 4.4.0) (R Core Team, 2022).

*Demographic and cognitive variables*: As all Montessori schools in Switzerland are private, *t*-tests were carried out to ensure that the groups were comparable. Age, gender and all cognitive and behavioral measures, such as socio-economic status, number of activities performed within the family, performance preoccupation, anxiety, emotion regulation (reappraisal and suppression), intelligence, executive functions (selective attention, working memory, switching) and creativity (divergent and convergent thinking) were assessed.

*FA, MD and ICVF relation to demographic and cognitive variables*: To assess the relationship between cerebellar white matter microstructure and behavioral measures, we performed Spearman’s rank correlation analyses between diffusion metrics of the four cerebellar tracts (left and right ICP, MCP, and SCP) and the set of cognitive and behavioral measures, including SES, pedagogy interest, number of activities performed within the family, parental worry about performance, anxiety, emotion regulation (reappraisal and suppression), intelligence, executive functions (selective attention, working memory, switching), and creativity (divergent and convergent thinking) scores. Spearman’s rho was chosen over Pearson’s r because several cognitive measures are derived from ordinal (Likert scale) data, which violates the continuity assumption of Pearson correlations. These analyses were conducted separately for the three diffusion metrics: FA, MD, and ICVF. Given the number of pairwise comparisons, Holm’s correction was applied to adjust for multiple testing and reduce the risk of Type I errors.

*Developmental trajectories and effect of school experience:* To analyze the relationship between cerebellar white matter microstructure and individual differences in pedagogy, age, gender, and SES, we employed different statistical models depending on the distributional properties of each diffusion metric. For mean diffusivity (MD), which follows a continuous unbounded distribution, we used MANCOVA with the four cerebellar peduncles (left and right ICP, MCP, and SCP) as dependent variables. For fractional anisotropy (FA) and intracellular volume fraction (ICVF), which are bounded between 0 and 1 and therefore better modelled with beta regression (Ferrari and Cribari-Neto, 2004) we fitted separate beta regression models per tract using maximum likelihood estimation (MLE) with a logit link. Predictor variables in all models included pedagogy (Montessori vs. Traditional), age, gender, and SES, with a pedagogy-by-age interaction term. For example, the MANCOVA model for mean diffusivity was specified as:

ICP_R_MD, ICP_L_MD, MCP_MD, SCP_MD ∼ pedagogy*age + gender + ses

For MD, multivariate significance was first assessed using Wilks’ lambda (Rao’s F-approximation). When a multivariate effect reached significance (p < .05), follow-up univariate F-tests identified which tracts drove the effect; multiple-comparison correction applied Holm’s method within the set of tracts tested. For FA and ICVF, statistical inference used z-tests derived from the expected Fisher information matrix of the beta regression models, with the same sequential Holm correction procedure. Prior to beta regression model fitting, boundary values were adjusted using the formula y* = [y(n - 1) + 0.5]/n (Smithson and Verkuilen, 2006).

Furthermore, to assess the potential of white matter microstructure metrics in classifying participants based on their pedagogical background (Traditional vs. Montessori), three receiver operating characteristic (ROC) curve analyses were performed. Specifically, we computed ROC curves to evaluate the classification accuracy using FA, ICVF, and MD values within each of the four cerebellar peduncles (left and right ICP, MCP, and SCP). For each model, a logistic regression was fitted using the respective metric as a predictor, and the area under the curve (AUC) was computed to quantify the classification performance. The ROC curves were plotted to visually inspect the sensitivity and specificity of each metric, with higher AUC values indicating better discrimination between students from the two pedagogical groups.

*Longitudinal data:* To assess differences in developmental change trajectories between students in Montessori and Traditional schooling, a linear mixed-effects (LME) framework was applied. For balanced two-timepoint data, LME is mathematically equivalent to an analysis of covariance (ANCOVA) of follow-up scores controlling for baseline (y_T2 ∼ baseline + Pedagogy + Age + Gender + SES), partitioning between-subject variance identically to a random subject intercept and affording greater statistical power than change-score analysis when baseline-follow-up correlations are high (Bernal-Rusiel et al., 2013). Given the small longitudinal sample (n = 34), inference was conducted non-parametrically: a permutation test (10,000 permutations) was used to build an empirical null distribution of the Pedagogy t-statistic, yielding permutation p-values (p_perm) free of distributional assumptions; a bootstrap procedure (10,000 resamples with replacement) provided 95% confidence intervals on the standardised Pedagogy coefficient.

## 3 Results

### 3.1 Demographics and cognitive variables

Statistical analysis revealed comparable and homogeneous groups in terms of age (M Montessori = 9.08, SD = 2.13, M Traditional = 9.19, SD = 2.29, U(86) = 959, *p* = 0.953) and number of girls and boys (Chi2 = 0.046, *p* = 0.831).

Independent *t*-tests were conducted to compare behavioral and cognitive measures between students in Traditional and Montessori pedagogies. No significant differences were observed in socioeconomic status (p = 0.992), pedagogy interest (M Traditional = 1.89, SE = 0.115; M Montessori = 2.10, SE = 0.098; p = 0.201), or activities in family (p = 0.366). However, parental worry about their child’s academic performance was significantly lower in the Montessori group (M = 5.12, SE = 0.478) compared to the Traditional group (M = 9.07, SE = 0.276), t(82) = -7.237, p < .001. Divergent thinking (M Montessori= 8.951, SE = 0.699 and M Traditional = 6.159, SE = 0.582, t(83) = 3.086, p = 0.00275) and convergent thinking (M Montessori = 4.862, SE = 0.250, M Traditional = 3.520, SE = 0.206, t(81) = 4.169, p < .0001) were also significantly higher in the Montessori group, suggesting potential advantages in creative and problem-solving skills. No significant differences were found in anxiety, emotional regulation (suppression and cognitive reappraisal), fluid intelligence, selective attention, working memory, or cognitive flexibility (all p > .05). These findings suggest that while most cognitive abilities were similar across pedagogies, Montessori students demonstrated lower parental worry and higher creative problem-solving abilities (see Table 1, Fig. 2B).

For the longitudinal subgroup (n = 34), demographic and cognitive characteristics at both time points are provided in Supplementary Table S1. At T1, the two pedagogy groups were comparable in age (t = 0.94, p = .355), sex (χ² = 0.18, p = .667), and SES (t = -0.83, p = .413). Consistent with the full cross-sectional sample, Montessori children in the longitudinal subsample scored significantly higher on divergent thinking (Montessori: 11.1 ± 4.0 vs. Traditional: 6.6 ± 4.8; t = -2.96, p = .006) and convergent thinking (4.3 ± 1.6 vs. 3.1 ± 1.4; t = -2.36, p = .025), whereas Traditional children showed higher anxiety percentile scores (36.4 ± 12.7 vs. 26.3 ± 14.5; t = 2.13, p = .042). Critically, no significant between-group differences were observed in any diffusion metric at T1 (all p > .29), ruling out pre-existing microstructural differences as a confound for the longitudinal effects. Paired comparisons across the full longitudinal subgroup confirmed robust age-related changes from T1 to T2: MD decreased significantly in all four cerebellar tracts (all p < .001) and ICVF increased significantly in all four tracts (all p < .001), consistent with continued myelination and axonal maturation. FA did not change significantly in any tract (all p > .13).

### 3.2 Cerebellar tracts

#### 3.2.1 FA, ICVF and MD relation to demographic and cognitive variables

Using Spearman’s rank correlation analyses (ρ), we examined the associations between cerebellar white matter microstructure and cognitive performance. To control for multiple comparisons, Holm’s family-wise correction was applied per cognitive variable across the four cerebellar tracts (4 tests per variable), testing whether each cognitive or behavioral measure showed consistent associations with cerebellar microstructure across tracts. Under this correction, one association survived: higher intracellular volume fraction (ICVF) in the right inferior cerebellar peduncle (ICP_R) was significantly associated with better selective attention performance (ρ = +0.311, p = .009, p_Holm =.038, n = 69). Further associations reached uncorrected p < .05 and are reported as exploratory trends pending replication in larger samples.

For ICVF, positive associations with fluid intelligence were observed in the left ICP (ρ = +0.22, p = .044) and SCP (ρ = +0.25, p = .022), with selective attention in the left ICP (ρ = +0.24, p = .046) and, as noted above, the right ICP (ρ = +0.31, p_Holm =.038), and with working memory in the MCP (ρ = +0.25, p = .036). For MD, negative associations with selective attention were observed in the right ICP (ρ = −0.26, p = .033) and left ICP (ρ = −0.27, p = .026), and with fluid intelligence in the SCP (ρ = −0.22, p = .040). The convergent direction of ICVF and MD findings, i.e., higher neurite density and lower diffusivity both indexing denser, more myelinated axonal tissue, strengthens the biological plausibility of these associations. Additionally, a positive association between the number of activities done in family and right ICP MD was observed (ρ = +0.22, p = .036). No associations reached uncorrected p < .05 for FA. All correlation values are illustrated in Fig. 2C.

### 3.3 Developmental trajectories and the effect of school experience

Associations between pedagogy (Traditional vs. Montessori) and cerebellar white matter microstructure were examined for three diffusion metrics (FA, ICVF, MD) across the four cerebellar peduncles (left and right ICP, MCP, and SCP), while controlling for age, gender, socioeconomic status (SES), and the pedagogy-by-age interaction. MANCOVA was used for MD, and beta regression (maximum likelihood estimation, logit link) for FA and ICVF.

**Fig. 3:**
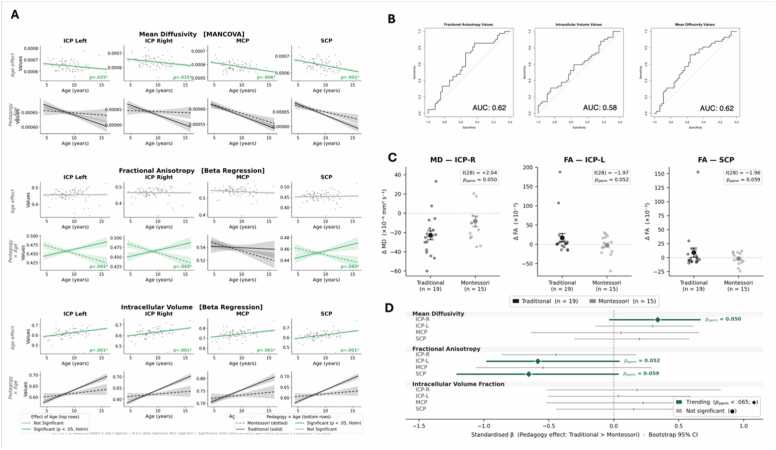
**Developmental trajectories, schooling effects, classification performance, and longitudinal changes in cerebellar white matter microstructure.** (A) Developmental trajectories alone and as a function of schooling experience (Montessori vs. Traditional) of all diffusion metrics (i.e., fractional anisotropy (FA), intra-cellular volume fraction (ICVF), and mean diffusivity (MD)) across the cerebellar peduncles (left and right inferior cerebellar peduncles, ICP; middle cerebellar peduncle, MCP, and superior cerebellar peduncle, SCP). Significant effects of age, as determined by MANCOVA analyses, are highlighted in green. Significant age × pedagogy interactions, as identified through beta regression analyses, are highlighted in green. (B) Receiver operating characteristic (ROC) curves assessing the ability of diffusion metrics (FA, ICVF, and MD) to classify participants into pedagogical groups (Montessori, traditional). The area under the curve (AUC) is reported for each metric, showing above-chance classification, albeit with modest effects. (C) Longitudinal LME analysis: For participants assessed at two time points over a mean interval of 2.9 years (N = 34; Montessori = 14, Traditional = 20), tract-specific linear models (*y*_T2∼ baseline + Pedagogy + Age + Gender + SES) were used to examine longitudinal changes in diffusion metrics. Inference was conducted using permutation testing and bootstrapped confidence intervals to account for the modest sample size. Trend-level pedagogy-related differences were observed in the inferior cerebellar peduncle (ICP) and superior cerebellar peduncle (SCP). Traditional-schooled participants showed a larger decrease in mean diffusivity (MD) in the right ICP and greater increases in fractional anisotropy (FA) in the left ICP and SCP compared to Montessori-schooled participants.

MANCOVA results for MD revealed no significant multivariate effect of pedagogy in any peduncle (all p > .19; multivariate: Wilks’ λ =.977, F(4, 77) = 0.45, p = .773). A significant multivariate Age effect was detected (Wilks’ λ =.843, F(4, 77) = 3.58, p = .010); Holm-corrected follow-up univariate tests confirmed significant age associations in all four peduncles: right ICP (p = .035), left ICP (p = .035), MCP (p = .006), and SCP (p = .002), indicating that mean diffusivity decreases with age across cerebellar white matter (see Fig. 3A). No significant effects were found for gender, SES, or the pedagogy-by-age interaction (all multivariate p > .22).

For FA, beta regression results indicated no main effect of pedagogy in any peduncle (all p > .18). However, significant Pedagogy × Age interactions were observed in the left ICP (p = .041), right ICP (p = .042), and SCP (p = .042), indicating that the developmental trajectory of FA differed between pedagogical groups in these tracts (see Fig. 3B). Age did not independently predict FA in any peduncle (all p > .26). No significant effects were found for gender or SES (all p > .10).

For ICVF, beta regression results revealed no significant main effect of pedagogy in any peduncle (all p > .07). Age was a significant predictor across all four peduncles: right ICP (p < .001), left ICP (p < .001), MCP (p = .001), and SCP (p < .001), reinforcing the broad influence of age on cerebellar microstructure (see Fig. 3B). The Pedagogy × Age interaction was not significant (all p > .21); gender and SES were also non-significant in all peduncles (all p > .06). Table 2 presents a detailed summary of the statistical results.

**Table 2:** Statistical results (Holm-corrected p-values) for pedagogy, age, gender, SES, and the pedagogy-by-age interaction across the four cerebellar peduncles for mean diffusivity (MD; MANCOVA, univariate follow-ups), fractional anisotropy (FA; beta regression, z-tests), and intracellular volume fraction (ICVF; beta regression, z-tests). All reported p-values are Holm-corrected within each metric. ICP_R = Right Inferior Cerebellar Peduncle, ICP_L = Left Inferior Cerebellar Peduncle, MCP = Middle Cerebellar Peduncle, SCP = Superior Cerebellar Peduncle.

**Region**	**Metric**	**Pedagogy (p-value)**	**Age (p-value)**	**Gender (p-value)**	**SES (p-value)**	**Pedagogy × Age (p-value)**
**ICP_R**	MD	0.768	**0.035**	1.000	1.000	0.516
**ICP_L**	MD	0.768	**0.035**	0.660	1.000	0.645
**MCP**	MD	0.657	**0.006**	1.000	1.000	0.649
**SCP**	MD	0.612	**0.002**	1.000	1.000	0.645
**ICP_R**	FA	1.000	1.000	1.000	1.000	**0.042**
**ICP_L**	FA	1.000	1.000	1.000	1.000	**0.041**
**MCP**	FA	1.000	1.000	1.000	1.000	0.374
**SCP**	FA	1.000	1.000	1.000	1.000	**0.042**
**ICP_R**	ICVF	0.340	**< 0.001**	0.414	1.000	0.114
**ICP_L**	ICVF	0.340	**< 0.001**	0.414	1.000	0.162
**MCP**	ICVF	0.340	**0.001**	0.414	1.000	0.162
**SCP**	ICVF	0.340	**< 0.001**	0.332	0.892	0.100

Receiver operating characteristic (ROC) curve analyses were performed to evaluate the ability of FA, ICVF, MD to classify participants based on their pedagogical background. The classification accuracy, as measured by the area under the curve (AUC), varied across the models. FA values within the four cerebellar peduncles yielded an AUC of 0.618 with an overall classification accuracy of 55.68%. MD values showed a slightly higher discriminative ability with an AUC of 0.623 and an accuracy of 59.09%. In contrast, ICVF values demonstrated the lowest classification performance, with an AUC of 0.575 and an accuracy of 53.41%, see Fig. 3C.

### 3.4 Longitudinal data

To examine whether white matter change over the three-year interval differed between pedagogy groups, an ANCOVA-equivalent LME model was fitted for each tract-metric combination (y_T2 ∼ baseline + Pedagogy + Age + Gender + SES; n = 34). Inference was conducted non-parametrically using permutation tests and bootstrapping to account for the small sample size. Three trend-level effects were observed. Traditional-schooled participants showed a larger decrease in mean diffusivity (MD) in the right ICP (ΔTrad = −23 × 10⁻⁶ mm² s⁻¹ vs. ΔMont = −8 × 10⁻⁶ mm² s⁻¹; t(28) = 2.04, p_perm = .050), directionally consistent with accelerated white matter maturation. In fractional anisotropy (FA), Traditional-schooled participants showed a larger increase in the left ICP (ΔTrad = +17.0 × 10⁻³ vs. ΔMont = −2.2 × 10⁻³; t(28) = -1.97, p_perm = .052) and SCP (ΔTrad = +8.9 × 10⁻³ vs. ΔMont = −2.0 × 10⁻³; t(28) = -1.96, p_perm = .059). Bootstrap 95% confidence intervals for all three effects included zero, indicating that the current sample size does not provide sufficient power to establish reliable Pedagogy effects longitudinally. These trends are directionally consistent with the cross-sectional age-by-pedagogy interactions. See Fig. 3D.

## 4 Discussion

The present study examined whether the developmental trajectories of cerebellar white matter pathways differ as a function of schooling experience. While the cerebellum is well established as a key contributor to motor control and higher-order cognition, its potential sensitivity to everyday learning environments has remained largely unexplored. Here, we provide evidence that the pace of cerebellar white matter maturation rather than absolute microstructural differences varies according to educational context. These effects were most prominent within the ICP, which support sensorimotor integration and learning, and the SCP, the principal efferent pathway linking the cerebellum to prefrontal and motor cortical regions. Together, these findings position schooling as a meaningful experiential factor associated with interindividual variability in cerebellar development.

The Montessori and traditional school groups were well matched in age and gender, minimizing potential confounds related to developmental stage or sex. Socioeconomic status and family activity levels, variables known to be associated with educational outcomes (Ogg and Anthony, 2020), were also comparable across groups, suggesting that the observed neurodevelopmental differences are unlikely to reflect broad socioeconomic disparities. Parental worry about academic performance was lower in the Montessori group, potentially reflecting absence of grading systems or test-based evaluation in Montessori pedagogy reducing parental stress about school outcomes, or a higher level of perceived child autonomy and academic engagement within Montessori environments. Anyhow, this variable did not account for the observed brain effects.

An important consideration when interpreting these findings concerns the self-selection mechanism inherent to Montessori schooling in Switzerland. Because all Montessori schools in the country are private, families who enroll their children in Montessori institutions are systematically different from those who do not. Relevant factors may include parental educational philosophy, personal values regarding child autonomy and creativity, prior beliefs about learning, and socioeconomic resources beyond those captured by the SES index. These family-level characteristics are difficult to disentangle from the pedagogical environment itself. Although we controlled for SES, we acknowledge that this covariate does not fully capture the constellation of environmental and familial factors that co-vary with school choice. Schooling context should therefore be understood as a proxy for a broader experiential environment, rather than as an isolated experimental manipulation. Future studies using longitudinal designs with pre-school measurements, or randomized school assignment where feasible, would be necessary to disentangle the specific contributions of pedagogy from pre-existing family-level characteristics.

At the behavioral level, children educated in Montessori settings showed higher performance on both divergent and convergent thinking measures, replicating prior reports of pedagogical influences on creativity (Denervaud et al., 2019). Divergent thinking is often associated with the capacity to generate novel ideas, while convergent thinking involves integration of information to arrive at a single outcome (Eon Duval et al., 2023). These findings are consistent with the idea that Montessori learning environments may be associated with differences in both creative ideation and structured problem-solving, while not permitting causal inference. However, no significant group differences were observed across a broad range of cognitive and affective domains, including fluid intelligence, attention, working memory, cognitive flexibility, anxiety, and emotion regulation. This relative behavioral equivalence across most domains strengthens the interpretation of the neuroimaging findings by reducing the likelihood that global cognitive differences account for the observed brain effects. Of note, it is possible that neurodevelopmental differences in cerebellar microstructure may precede or develop independently of detectable behavioral changes; that is, structural brain differences may emerge from differences in experience without necessarily manifesting as measurable performance differences within the timeframe of our assessment. This interpretation would be consistent with theories of neural efficiency and cerebellar internal models, whereby differences in the tuning of predictive circuits may be subtle at the behavioral level but still detectable at the microstructural level.

Across participants, cerebellar white matter microstructure was meaningfully related to cognitive performance. Higher ICVF within the ICP, MCP, and SCP was associated with better fluid intelligence, selective attention, and working memory, while lower MD values within ICP and SCP, were linked to enhanced selective attention and fluid intelligence. These associations are consistent with greater microstructural organization or cellular density supporting more efficient cognitive processing (Acosta-Rodriguez et al., 2024, Urbini et al., 2025). Interestingly, fluid intelligence was primarily related SCP, the main cerebellar output pathway to prefrontal and associative cortical regions, consistent with its role in higher-order reasoning and cognitive control. In contrast, selective attention was most strongly linked to the ICP, which conveys afferent sensory information and may support attentional selection through rapid integration and prediction of incoming signals. Working memory was associated with MCP, reflecting its role in transmitting cortical inputs to the cerebellum and supporting information maintenance and integration. Together, this pattern suggests a functional dissociation between afferent (ICP), integrative (MCP), and efferent (SCP) pathways. However, these relationships are correlational and do not imply causal directionality, but they align with developmental models emphasizing the cerebellum’s contribution to cognitive efficiency (Mastrangelo et al., 2024, Tiemeier et al., 2010, Travis et al., 2015, Zhang et al., 2023). FA was not associated with cognitive or demographic measures, suggesting that FA may be less sensitive to individual differences in this age range.

Consistent with prior developmental neuroimaging studies (Lebel et al., 2012, Olson et al., 2023), age was a strong predictor of MD and ICVF across all cerebellar peduncles, reflecting prolonged microstructural maturation throughout childhood and adolescence. In contrast, FA did not show significant age-related effects, mirroring previous reports that FA may plateau or show reduced sensitivity during certain developmental windows in some tracts (Bonekamp et al., 2007, Johnson et al., 2014). These findings further support the notion that cerebellar white matter maturation extends well into adolescence and early adulthood (Lebel et al., 2012).

Crucially, while pedagogy did not predict absolute diffusion metrics, significant ageXpedagogy interactions indicate that the rate of cerebellar maturation differs across schooling contexts. Children educated in Montessori settings exhibited relatively stable FA values across age within the ICP and SCP, whereas children in traditional schools showed steeper age-related increases in these metrics. The finding that FA increases more rapidly with age in traditionally schooled children is notable: FA reflects directional coherence of water diffusion in white matter, often interpreted as an index of myelination and axonal organization (Beaulieu, 2002). One interpretation is that the more structured and repetitive sensorimotor demands of traditional schooling, such as handwriting, seating posture, and teacher-led activities, may drive more rapid consolidation of specific cerebellar-cortical circuits. Conversely, the more flexible and autonomy-based sensorimotor environment of Montessori schooling may be associated with a more gradual but broader tuning of these circuits. However, the directionality of these differences, which developmental trajectory is more advantageous, cannot be determined from the current data, and both may represent adaptive responses to distinct learning demands. Importantly, these associations reflect differences in developmental timing rather than categorical group differences at any given age. Such divergence in maturation slopes suggests that schooling environments may modulate how cerebellar circuits are refined over time. Furthermore, while no longitudinal Pedagogy effects were significant, three trend-level effects (all p_perm =.050–.059; bootstrap 95% CIs include zero) were directionally consistent with the cross-sectional patterns: traditionally-schooled children showed numerically larger microstructural change in the right ICP (MD) and in the left ICP and SCP (FA). The directional concordance between cross-sectional and longitudinal patterns lends descriptive support to the interpretation of differential developmental tempos, though these longitudinal trends should be interpreted with caution given the modest sample size (n = 34). Given the ICP’s role in integrating somatosensory input and supporting adaptive motor-cognitive coordination, these findings may reflect differences in movement, feedback frequency, and task structure embedded in distinct learning environments. Past work report how Montessori vs traditionally-schooled students deploy different multisensory competences (Denervaud et al., 2020b). Similarly, the SCP that links cerebellar output to thalamic and prefrontal circuits, plays a key role in executive control and planning. This apparent difference in developmental tempo, with Montessori-schooled children showing a more gradual rate of microstructural change, may reflect extended tuning of cerebellar-cortical loops in learning environments characterized by greater autonomy and self-regulation, potentially in interaction with reduced evaluative pressure (Moreno-Rius, 2019, Sisk and Gee, 2022). It should be noted that this interpretation remains speculative, as the data are partly cross-sectional and the longitudinal window covers a single interval. This “protracted maturation”, descriptively indicating a slower rate of change, not to assert a definitive endpoint, may reflect the cerebellum’s sensitivity to stress and environmental demands (Moreno-Rius, 2019, Sisk and Gee, 2022).

Taken together, these findings contribute to a growing literature demonstrating that environmental experiences shape not only cognitive outcomes but also underlying neurodevelopmental trajectories (Gee, 2022). Rather than uniformly accelerating maturation, learning environments characterized by differing cognitive, motor, and social demands may be associated with distinct developmental tempos, supporting experience-dependent plasticity in cerebellar circuits. Despite these developmental differences, diffusion metrics alone showed limited ability to classify individuals based on schooling context, with modest AUC values across FA, MD, and ICVF. This suggests that cerebellar microstructure does not encode educational background in a deterministic manner, but rather reflects one component of a broader, distributed system underlying individual variability in development. Such findings reinforce the view that schooling-related neural effects are probabilistic and multifactorial.

However, several limitations should be considered when interpreting these findings. First, although the study combines cross-sectional and longitudinal data, the design remains observational, preventing causal inference regarding the associations between schooling context and cerebellar development. Schooling experience likely co-varies with unmeasured environmental factors, such as classroom practices, teacher behaviors, or extracurricular activities, which were not directly quantified here. Second, the longitudinal subsample was modest in size, limiting statistical power to detect subtle developmental effects and precluding more fine-grained modeling of individual growth trajectories. Third, pedagogical context was treated as a categorical variable, whereas educational environments are inherently heterogeneous. Furthermore, the self-selection process inherent to Montessori enrollment, whereby families choose this pedagogy based on their values, beliefs, and financial resources, means that schooling context likely reflects a constellation of correlated environmental, parenting, and cultural factors beyond pedagogy per se. Although SES was controlled for, it does not fully account for the range of family-level differences that may co-vary with school choice. Future work would benefit from prospective designs with baseline measurements prior to school enrollment, random assignment where feasible, continuous measures of classroom dynamics, autonomy, movement, and evaluative pressure, and more fine-grained characterization of family-level factors. Fourth, while diffusion metrics such as FA, MD, and ICVF provide sensitive markers of white matter microstructure, they remain indirect proxies of underlying cellular processes and should not be interpreted as specific measures of myelination or axonal density. Finally, the present sample comes from a specific cultural and educational context, which may limit generalizability to other schooling systems or sociocultural settings. Addressing these limitations in larger, multi-site longitudinal studies integrating behavioral, environmental, and neurobiological measures will be essential for refining models of experience-dependent cerebellar development.

In conclusion, this study provides preliminary but convergent evidence that cerebellar white matter development follows experience-sensitive trajectories associated with schooling context. While pedagogy did not predict absolute microstructural differences, age-dependent modulation of cerebellar peduncles highlights the cerebellum’s sensitivity to everyday learning environments. These results underscore the importance of incorporating environmental variables into developmental neuroscience models and suggest that schooling experiences contribute to shaping variability in brain maturation across childhood and adolescence.

## CRediT authorship contribution statement

**Solange Denervaud:** Writing – original draft, Visualization, Validation, Supervision, Project administration, Investigation, Funding acquisition, Formal analysis, Data curation, Conceptualization. **Fischi Gomez Elda:** Writing – review & editing, Validation, Methodology, Conceptualization. **Mathilde Gaujard:** Writing – original draft, Supervision, Investigation, Formal analysis. **Gabriel Girard:** Writing – review & editing, Visualization, Validation, Methodology, Formal analysis, Conceptualization. **Camille Grosjean:** Writing – original draft.

## Institutional Review Board Statement

This study was approved by the local ethics commission (CER-VD).

## Declaration of Generative AI and AI-assisted technologies in the writing process

During the preparation of this work the authors used ChatGPT to correct and improve English grammar. After using this tool/service, the authors reviewed and edited the content as needed and take full responsibility for the content of the published article.

## Declaration of Competing Interest

The authors declare no competing interests.

## Data Availability

Data will be made available on request.

## Footnotes

Appendix A Supplementary data associated with this article can be found in the online version at doi:10.1016/j.dcn.2026.101748.
