# Supplementary material for: When little brain goes to school: Impact of pedagogy on cerebellar peduncles’ development
Source: Dev Cogn Neurosci. 2026 May 25;80:101748. doi: 10.1016/j.dcn.2026.101748 (PMC13234714; doi:10.1016/j.dcn.2026.101748)
Supplement: Supplementary file 1 — Supplementary material [file mmc1.pdf]

Supplementary Table S1. Demographic, cognitive, and diffusion characteristics of the longitudinal subgroup by pedagogy group at T1 and T2.

	**T1**				**T2**			
**Variable**	**Montessori (n = 15)**	**Traditional (n = 19)**	**t / χ^2**	**p**	**Montessori (n = 15)**	**Traditional (n = 19)**	**t / χ^2**	**p**
**DEMOGRAPHIC & COGNITIVE VARIABLES**								
Age (years)	8.91 ± 1.51	9.48 ± 2.02	0.94	.355	11.77 ± 1.53	12.35 ± 2.11	0.92	.366
Girls, n (%)	6 (40%)	9 (47%)	0.18	.667	6 (40%)	9 (47%)	—	—
SES	3.20 ± 0.49	3.05 ± 0.49	−0.83	.413	3.28 ± 0.57	3.08 ± 0.63	−0.99	.329
Pedagogy interest	2.57 ± 0.51	2.37 ± 0.83	−0.86	.394	2.87 ± 0.35	2.50 ± 0.71	−1.93	.064
Family activities	9.21 ± 1.81	9.44 ± 1.46	0.39	.701	6.93 ± 2.94	6.26 ± 1.82	−0.77	.447
Worry about performance	0.61 ± 0.36	0.87 ± 0.21	**2.41**	**.026**	0.57 ± 0.35	0.92 ± 0.19	**3.49**	**.002**
Anxiety (%)	26.33 ± 14.54	36.45 ± 12.70	**2.13**	**.042**	34.81 ± 12.94	39.33 ± 11.27	1.07	.295
Fluid intelligence	32.93 ± 4.08	31.74 ± 3.98	−0.86	.398	34.40 ± 2.80	34.11 ± 1.70	−0.36	.723
Divergent thinking	11.07 ± 3.97	6.56 ± 4.77	**−2.96**	**.006**	8.07 ± 2.89	6.47 ± 3.20	−1.52	.138
Convergent thinking	4.33 ± 1.55	3.11 ± 1.39	**−2.36**	**.025**	5.53 ± 1.03	4.49 ± 1.00	**−2.96**	**.006**
ER: Reappraisal	18.53 ± 5.67	15.32 ± 5.01	−1.73	.095	19.93 ± 4.48	19.21 ± 5.15	−0.43	.672
ER: Suppression	7.20 ± 3.57	8.63 ± 4.92	0.98	.334	8.86 ± 3.80	6.53 ± 2.46	−2.01	.058
**DIFFUSION METRICS (M ± SD)**								
MD values are expressed as $\times 10^{-4}$ mm^2/s. FA and ICVF are unitless (range 0–1).								
**ICP-R**								
FA	0.467 ± 0.017	0.462 ± 0.046	−0.44	.667	0.468 ± 0.031	0.483 ± 0.025	1.51	.143
MD	6.68 ± 0.49	6.36 ± 0.53	−0.79	.437	6.60 ± 0.38	6.38 ± 0.42	−1.69	.100
ICVF	0.619 ± 0.044	0.634 ± 0.034	1.07	.296	0.650 ± 0.035	0.653 ± 0.031	0.28	.781
**ICP-L**								
FA	0.458 ± 0.021	0.458 ± 0.039	0.05	.960	0.455 ± 0.035	0.475 ± 0.025	1.85	.077
MD	6.83 ± 0.49	6.42 ± 0.62	−0.21	.839	6.59 ± 0.35	6.43 ± 0.59	−1.01	.321

	T1				T2			
**Variable**	**Montessori (n = 15)**	**Traditional (n = 19)**	**t / χ²**	**p**	**Montessori (n = 15)**	**Traditional (n = 19)**	**t / χ²**	**p**
ICVF	0.613 ± 0.048	0.623 ± 0.030	0.68	.501	0.641 ± 0.038	0.649 ± 0.032	0.60	.555
**MCP**								
FA	0.539 ± 0.020	0.541 ± 0.038	0.16	.871	0.535 ± 0.023	0.548 ± 0.022	1.62	.116
MD	6.36 ± 0.28	6.07 ± 0.36	−0.97	.343	5.96 ± 0.25	5.80 ± 0.33	−0.85	.400
ICVF	0.729 ± 0.054	0.742 ± 0.037	0.75	.463	0.775 ± 0.044	0.776 ± 0.034	0.08	.937
**SCP**								
FA	0.454 ± 0.018	0.457 ± 0.028	0.40	.692	0.452 ± 0.018	0.466 ± 0.017	**2.33**	**.027**
MD	6.73 ± 0.42	6.40 ± 0.44	−0.97	.343	6.40 ± 0.33	6.18 ± 0.34	−1.52	.140
ICVF	0.615 ± 0.041	0.623 ± 0.026	0.70	.489	0.653 ± 0.039	0.657 ± 0.030	0.35	.732

Note. Values are M ± SD unless otherwise indicated. T1 = baseline assessment; T2 = follow-up (~3 years later). Montessori n = 15, Traditional n = 19. Between-group comparisons used Welch's independent-samples t-tests; sex was compared using a chi-square test (same participants at both time points, so sex does not differ). Bold p-values indicate $p < .05$. FA = fractional anisotropy; MD = mean diffusivity; ICVF = intracellular volume fraction; ICP-R/L = right/left inferior cerebellar peduncle; MCP = middle cerebellar peduncle; SCP = superior cerebellar peduncle; ER = emotion regulation.
